# Editorial: Nutrition and neurobehaviors

**DOI:** 10.3389/fnut.2025.1637728

**Published:** 2025-06-17

**Authors:** Lina Begdache, Nazli Nur Aslan Çin, Nafisa M. Jadavji

**Affiliations:** ^1^Department of Health and Wellness Studies, Binghamton University, Binghamton, NY, United States; ^2^Department of Nutrition and Dietetics, Faculty of Health Sciences, Karadeniz Technical University, Trabzon, Türkiye; ^3^Department of Biomedical Sciences, School of Medicine, Southern Illinois University, Carbondale, IL, United States; ^4^Department of Child Health, College of Medicine-Phoenix, University of Arizona, Phoenix, AZ, United States; ^5^Department of Neuroscience, Carleton University, Ottawa, ON, Canada

**Keywords:** nutrition, behavior, cognitive function, anxiety, brain health

The interplay between nutrition and brain health has become a critical area of research. Examining how dietary choices impact cognitive function, mental wellbeing, and the risk profile of neurodegenerative diseases (1). A growing body of evidence demonstrates that diet significantly influences biochemical pathways regulating brain structure and function. Several nutrients play pivotal roles in neuroprotection, synaptic plasticity, and the mitigation of oxidative stress and inflammation, which are central to neurodegenerative processes Picone et al. (2). Therefore, diet quality and/or nutrient deficiency can affect brain function at different levels: neurotransmission, homeostasis, neuroinflammation, neurodegeneration, and neurodevelopment, among many more.

Studies included in our Nutrition and Neurobehaviors Research Topic highlight the critical interplay between nutrition and brain health, emphasizing the role of diet in cognitive function, mental wellbeing, and neurodegenerative disease risk. Evidence shows that high diet quality modulates biochemical pathways regulating brain structure and function. With nutrients like omega-3 fatty acids, antioxidants, vitamins, and minerals playing essential roles in neuroprotection, synaptic plasticity, and mitigating oxidative stress and inflammation, which are key contributors to the neurodegenerative processes Picone et al. (2).

The Mediterranean diet, abundant in fruits, vegetables, whole grains, and healthy fats, is rich in antioxidants, vitamins, and minerals that neutralize free radicals linked to neuronal degeneration Picone et al. Bioactive compounds present in the Mediterranean diet, such as polyphenols and omega-3 fatty acids, contribute to epigenetic mechanisms like DNA methylation and histone modification, reducing inflammation and activating antioxidant pathways (3). These compounds also modulate the gut-brain axis, a bidirectional communication system influenced by diet. A healthy, fiber-rich gut microbiota enhances cognitive function and emotional resilience through neural, endocrine, and immune interactions (4). For instance, Huang et al. reported that individuals with suicidal ideation had lower fiber intake, suggesting that dietary fiber may influence suicidal thoughts by modulating the gut microbiota's microbial composition and enhancing serotonin synthesis. Serotonin, a critical neurotransmitter in emotional regulation, is associated with an increased risk of suicidal behavior at low levels (5). Additionally, short-chain fatty acids (SCFAs), produced from dietary fiber fermentation, can inhibit histone deacetylases, induce epigenetic modifications, and upregulate brain-derived neurotrophic factor (BDNF) levels, potentially reducing suicide risk (6).

Conversely, Western diets high ultra-processed foods and refined sugars, disrupt the gut microbiota, potentially exacerbating neuroinflammation and psychosis. Kennedy et al. reported that individuals with psychosis consumed diets higher in processed carbohydrates and refined sugars, which may destabilize the gut microbiome and increase insulin resistance Kennedy et al. (7). Anti-inflammatory diets rich in omega-3 fatty acids, cruciferous vegetables, and probiotics and low in processed carbohydrates and refined sugars have been shown to regulate metabolic processes through SCFA production, which is commonly disturbed in psychiatric disorders Kennedy et al. (8). SCFAs exert their anti-inflammatory effects and improve intestinal barrier function through different mechanisms, such as pH modulation and activating G-protein-coupled receptors, which enhance the function of the tight junctions (9, 10). Serum vitamin D levels were shown to be inversely associated with anxiety risk in US adults Wen et al. as well as with sleep disorders Arabshahi et al..

Functional foods such as royal jelly (RJ) provide neuroprotective characteristics such as anti-inflammatory and antioxidant properties and enhance brain function by promoting hippocampal granule cell regeneration (11). Similarly, Liu J. et al. reported that elderly stroke patients with lower geriatric nutritional risk index scores were more likely to develop post-stroke cognitive impairment. In this context, RJ supplementation post-stroke is considered a potential alternative intervention for mitigating complications (Karimi et al.). The neuroprotective effects of RJ are mediated by the activation of AMP-activated protein kinase (AMPK), which suppresses apoptotic, inflammatory, and oxidative pathways, including microglial inflammation. Furthermore, RJ consumption is hypothesized to enhance the production of neurotrophins such as BDNF and nerve growth factor (NGF), promoting synaptogenesis, neurogenesis, and acetylcholine production (12). Both studies advocate for a delicate approach to nutritional research, recognizing that factors such as health status, sex-specific differences, and nutrient interactions play crucial roles in mental health outcomes.

The relationship between diet and anxiety is complex and influenced by multiple interconnected factors (13–16). Recent research highlights the pivotal role of the gut microbiome as a key mediator of mental health. Nutrient-dense diets provide antioxidants, prebiotics, and probiotics essential for brain and gut health. Western diets are associated with increased anxiety-like behaviors, potentially due to their contribution to stress (Basso et al.). A primary mechanism underlying this relationship appears to be the pro-inflammatory nature of these foods inducing dysbiosis, and amplifying the stress response. The lack of fiber and other prebiotic agents reduces beneficial butyrate-producing bacteria such as Faecalibacterium and Roseburia, which are known to mitigate stress-related mood decline. Butyrate, a short-chain fatty acid, supports colonic health and inflammation management, potentially contributing to its anxiolytic effects. Additionally, a high intake of added sugars promotes an increase in the Firmicutes/Bacteroidetes ratio while reducing butyrate-producing bacteria. Likewise, trans-fatty acids and processed meats negatively impact microbial diversity, further reinforcing the connection between unhealthy dietary choices and a compromised gut microbiome.

In contrast, diets rich in fruits, vegetables, and fiber promote beneficial gut bacteria, reducing inflammation and enhancing gut health. Individuals consuming a high intake of fruits and vegetables exhibit greater *Faecalibacterium* abundance compared to those in a placebo group. Similarly, fermented vegetable consumption has been linked to increased levels of *Faecalibacterium prausnitzii* and *Roseburia faecis*. Whole-grain intake also positively influences these beneficial bacteria. Rodríguez-Rangel et al. linked microbiota imbalances to binge-type eating behaviors and heightened susceptibility to anxiety-like behaviors. Research on specific nutrients such as omega-3 fatty acids, has yielded mixed results regarding anxiety outcome. Discrepancies may stem from variations in study design, sample size, and the type of omega-3 fatty acids examined. Additionally, factors such as age, sex, and overall health status may mediate these effects, stressing the need for personalized research approaches. Notably, sex-specific effects have been observed, with dietary components and gut bacteria exerting different effects on males and females. For example, a positive association exists between red meat consumption and anxiety in females but conflicting results in males. Moreover, specific gut bacteria exhibit sex-dependent associations with anxiety symptoms, further emphasizing the complexity of this relationship.

Gut microbiome research also extends to postpartum depression (PPD). Abdulrahim et al. highlight the impact of early microbiome establishment on maternal mental health. The observed decline in exclusive breastfeeding (EBF) rates among mothers with PPD may be exacerbated by dietary factors that disrupt the gut microbiome, leading to decreased levels of beneficial bacteria known to mitigate stress-related mood decline. In regions where access to nutrient-dense foods is limited, mothers may be particularly vulnerable to gut dysbiosis, worsening PPD symptoms (18). Furthermore, adopting intuitive and mindful eating habits have been reported to reduce mental distress (Acik and Aslan Cin).

Cognitive function refers to mental processes such as memory, attention, learning and problem-solving. Studies have identified that impairments in cognitive function most often leads to mild cognitive impairment (MCI) and eventually dementia (17). Studies by Gabriel Garrido-Dzib et al., Zhang et al., and Jiang et al. published in our Research Topic highlighted the importance of diet on cognitive function. More specifically, a study conducted in Yucatan, Mexico reported that the dietary pattern of individuals with mild cognitive impairment (MCI) and dementia have nutritional deficiencies. The findings recommended that adequate dietary intake of vegetables, fruits, and protein could improve the quality of life of patients with cognitive impairment (Gabriel Garrido-Dzib et al.). Furthermore, a study in middle-aged and elderly people in China found that berries, grapes, and red wine consumption can protect the basic cognitive state of the middle-aged and elderly, and the protective function is related to the Jiang et al.. Food security for cognitive health in the elderly is important to enhance cognitive functioning (Zhang et al.).

Our Research Topic also included studies addressing the role of specific nutrients on cognitive function. For instance, creatine monohydrate supplementation may confer beneficial effects on cognitive function in adults, particularly in the domains of memory, attention time, and information processing speed (Xu et al.). In addition, an optimal concentration range for serum levels of trimethylamine N-oxide (TMAO), betaine, and carnitine t mitigates MCI risk, paving the way for enhanced dietary interventions aimed at preventing and treating MCI (Bai et al.). Vitamin D has been shown to play an important role in cognitive function along with physical activity (Guo et al.). Larger, robust clinical trials are warranted to further validate these findings.

Lastly, remnant cholesterol (RC) levels and lower levels of TC total cholesterol (TC)/RC are associated with an increased likelihood of cognitive impairment, suggesting that RC can serve as a novel and convenient indicator for predicting the risk of cognitive impairment in the US population (Liu K. et al.). In female Parkinson's patients, there is a positive correlation between BMI and cognitive impairment, while no correlation was found in male patients. The role of sex differences in the relationship between BMI and cognitive impairment should be considered in future research (Wang et al.).

Despite promising evidence, several challenges remain. The complexity of dietary patterns, individual variability in nutrient metabolism, and the impact of socioeconomic factors on food accessibility complicate the translation of research findings into practical recommendations. Future studies should focus on elucidating the molecular mechanisms underlying the effects of specific nutrients on brain health, as well as developing personalized nutritional strategies tailored to individual genetic, metabolic, and lifestyle factors.
